# Supplementary material for: Evaluating impacts of syntenic block detection strategies on rearrangement phylogeny using Mycobacterium tuberculosis isolates
Source: Bioinformatics. 2023 Jan 13;39(1):btad024. doi: 10.1093/bioinformatics/btad024 (PMC9887090; doi:10.1093/bioinformatics/btad024)
Supplement: btad024_Supplementary_Data [file btad024_supplementary_data.pdf]

Supplementary Material

1 Supplementary Tables and Figures

Program	Version	Runtime
Hybran (annotation)	1.2.0	146 minutes
Hybran (annotation-relaxed)		100 minutes
SibeliaZ-LCB	1.2.2	3 minutes
Cactus	1.3.0	22 hours 17 minutes
DING	2021-01-26	2 hours 17 minutes (SibeliaZ-LCB) -<br>11 hours, 55 minutes (Cactus(Mash)-filtered)
Mash	2.3	
maf2synteny	1.0	
MLGO	1.0	1 minute (Cactus(SNP)-filtered+maf2synteny) -<br>12 minutes (Cactus(SNP))
FastME	2.1.6.2	

Table S1: Versions and running time for software used in this study

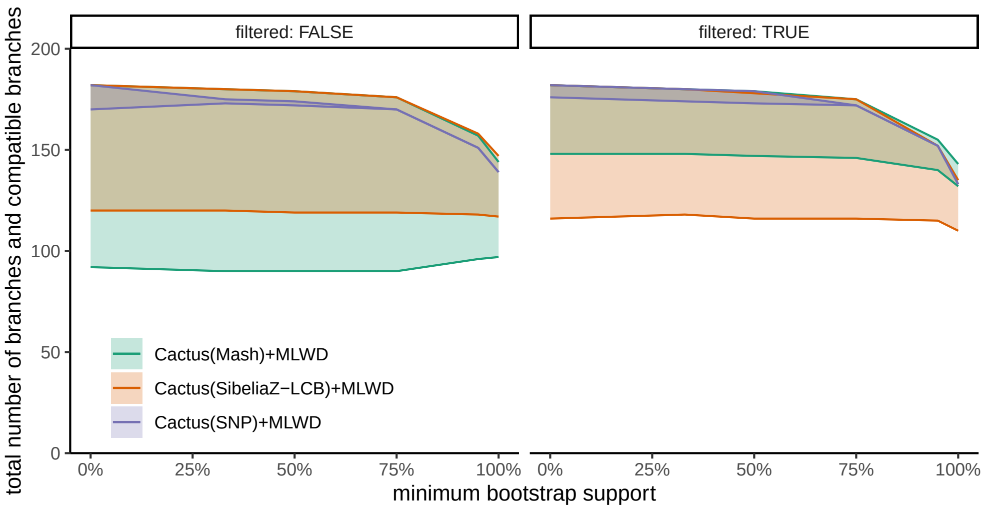

Figure S1: Compatibility of Cactus (with alignment guide tree indicated in parentheses) adjacency trees to the reference SNP tree before and after excluding blocks with fewer than 50 sites. Filtering of small blocks introduces a substantial improvement for Cactus(Mash) and more minimal differences for the alignments based on the other two guide trees.

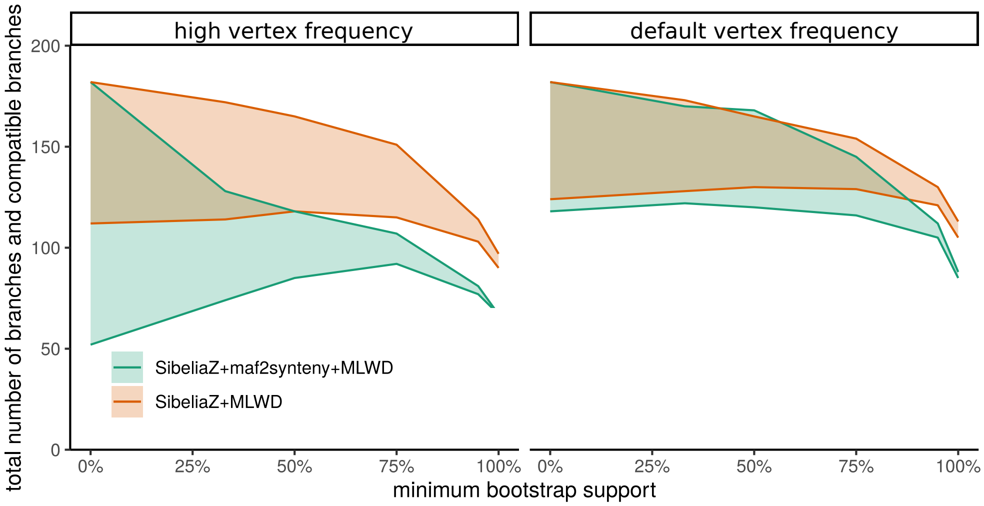

Figure S2: Compatibility of SibeliaZ adjacency trees to the reference SNP tree with and without raising the vertices frequency to allow delineation of more duplicated synteny blocks. Resolution and compatibility are uniformly lower for the high vertex frequency run (except at ≥50% bootstrap support on the blocks aggregated with maf2synteny).

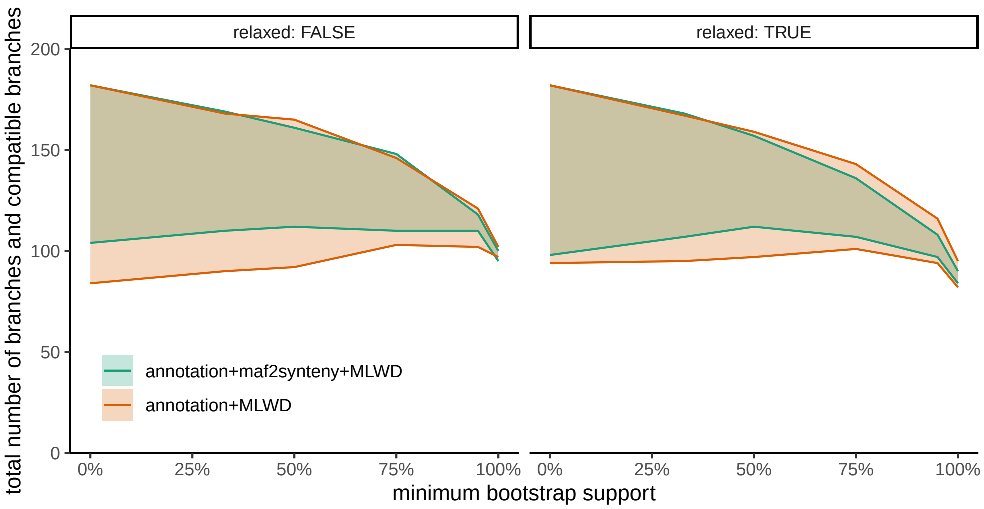

Figure S3: Compatibility of the annotation adjacency trees to the reference SNP tree based on default orthology-mapping parameters (minimum identity 95%, minimum alignment coverage 95%) and relaxed (minimum identity 75%, minimum alignment coverage 66%).

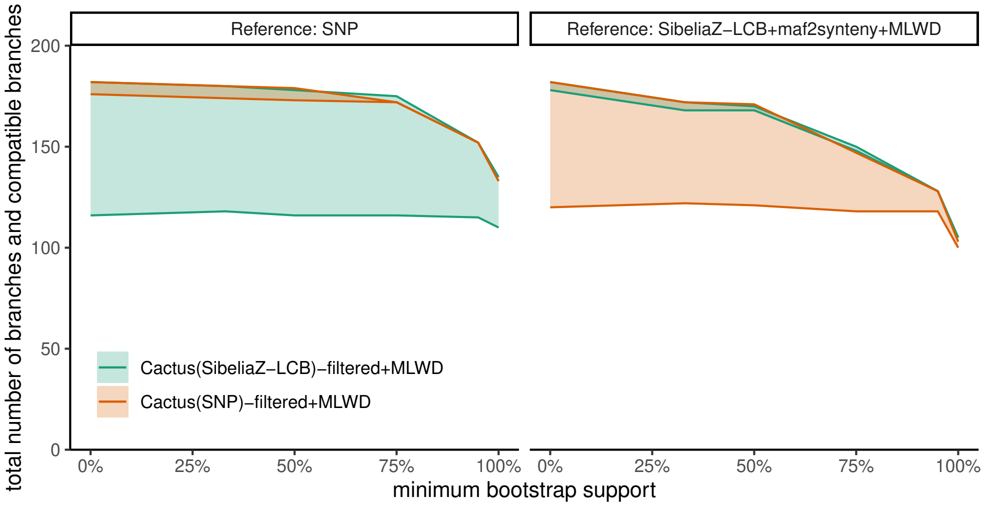

Figure S4: Compatibility of Cactus adjacency trees with different reference trees. Adjacency trees of Cactus alignment exhibit striking degrees of compatibility to the guide tree that was used to create the alignment and much less so to an alternative reference tree, even for branches with high BS.

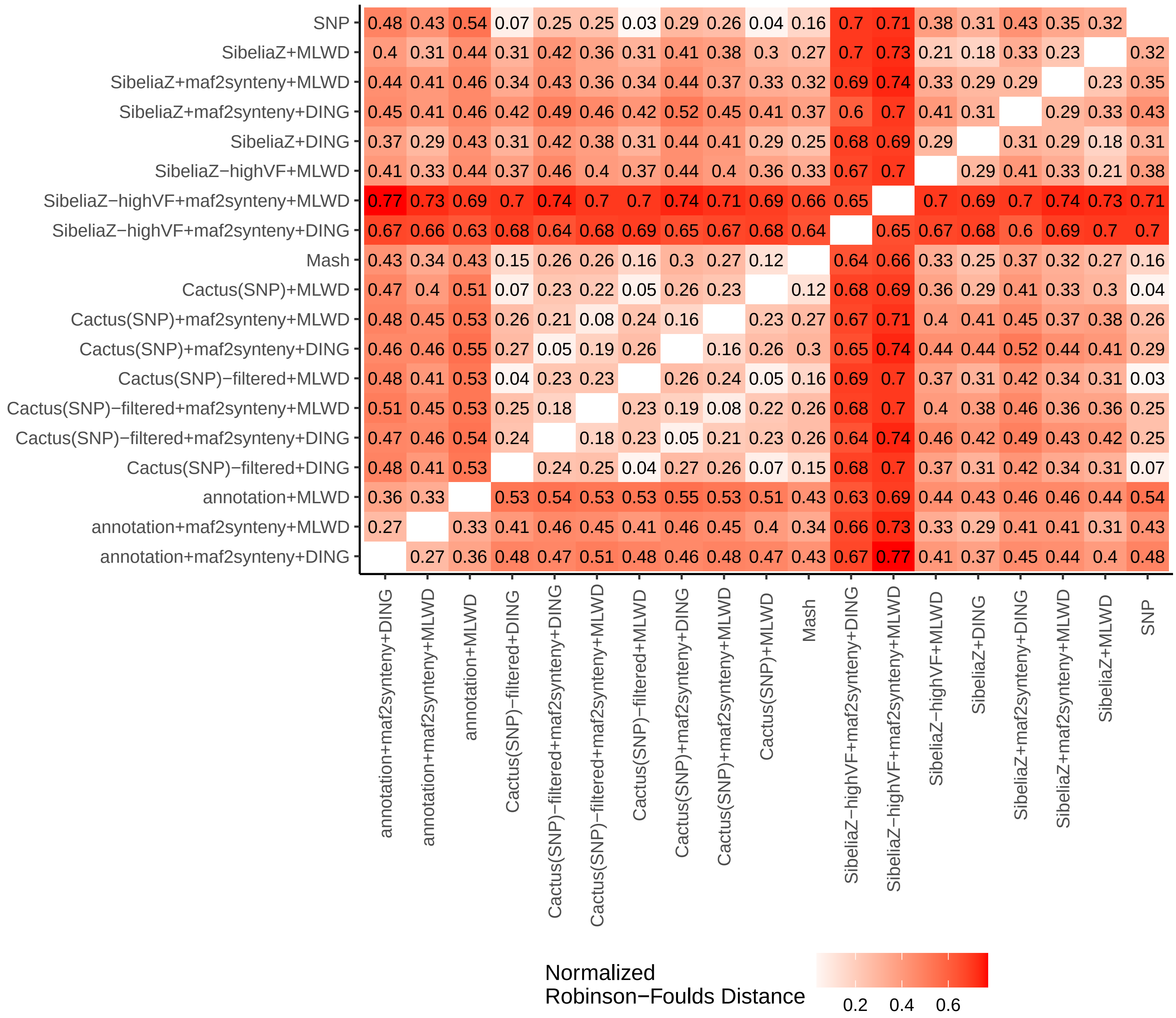

Figure S5: Normalized Robinson-Foulds distances

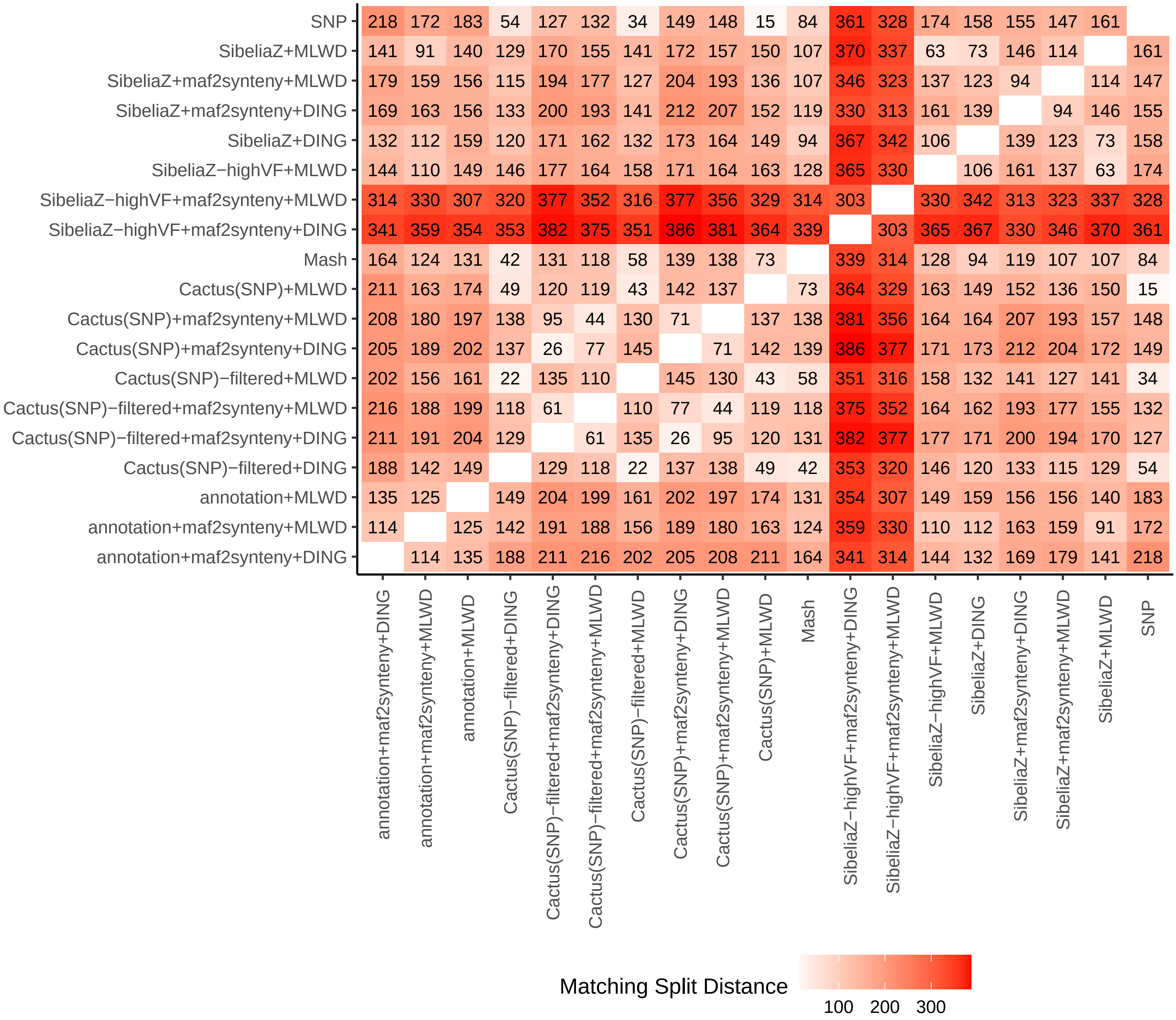

Figure S6: Matching split distances
